# Does the single-item self-rated health measure the same thing across different wordings? Construct validity study

**DOI:** 10.1007/s11136-020-02533-2

**Published:** 2020-05-20

**Authors:** Stéphane Cullati, Naike Bochatay, Clémentine Rossier, Idris Guessous, Claudine Burton-Jeangros, Delphine S. Courvoisier

**Affiliations:** 1 Population Health Laboratory, University of Fribourg, Fribourg, Switzerland; 2 Institute of Sociological Research, University of Geneva, Geneva, Switzerland; 3 Department of Readaptation and Geriatrics, University of Geneva, Geneva, Switzerland; 4 Quality of Care Service, Department of Readaptation and Geriatrics, Faculty of Medicine, University of Geneva, Chemin Thury 3, 1206 Geneva, Switzerland; 5 Department of Paediatrics, University of California, San Francisco, CA USA; 6 Unit of Development and Research in Medical Education, Faculty of Medicine, University of Geneva, Geneva, Switzerland; 7 Institute of Demography and Socioeconomics, University of Geneva, Geneva, Switzerland; 8 Division of Primary Care Medicine, Department of Primary Care Medicine, Geneva University Hospitals, Geneva, Switzerland

**Keywords:** Self-rated health, Questions wording, Response options, Construct validity, Population health measurement

## Abstract

**Purpose:**

The self-rated health (SRH) item is frequently used in health surveys but variations of its form (wording, response options) may hinder comparisons between versions over time or across surveys. The objectives were to determine (a) whether three SRH forms are equivalent, (b) the form with the best construct validity and (c) the best coding scheme to maximize equivalence across forms.

**Methods:**

We used data from 58,023 respondents of the Swiss Health Survey. Three SRH forms were used. Response options varied across forms and we explored four coding schemes (two considering SRH as continuous, two as dichotomous). Construct validity of the SRH was assessed using 34 health predictors to estimate the explained variance.

**Results:**

Distributions of response options were similar across SRH forms, except for the “good” and “very good” options (“good” in form 1: 58.6%, form 2: 65.0% and form 3: 44.1%). Explained variances differed across SRH forms, with form 3 providing the best overall explained variance, regardless of coding schemes. The linear coding scheme maximised the equivalence across SRH forms.

**Conclusion:**

The three SRH forms were not equivalent in terms of construct validity. Studies examining the evolution of SRH over time with surveys using different forms should use the linear coding scheme to maximise equivalence between SRH forms.

**Electronic supplementary material:**

The online version of this article (10.1007/s11136-020-02533-2) contains supplementary material, which is available to authorized users.

## Key-points

The self-rated health (SRH) item is frequently used in health surveys but variations of its form (wording, response options) may hinder comparisons between versions over time or across surveys

SRH forms were not equivalent in terms of construct validity

Linear coding scheme of the SRH maximises the equivalence between forms.

## Introduction

The self-rated health (SRH) item (also called self-assessed health or self-perceived health) is frequently used in demographic and population health surveys to capture respondents’ self-reported general health. The success of this self-reported indicator is explained by its ease of use (a single question), its validity [1, 2] and reliability [3, 4]. Particularly, the SRH item predicts mortality [5, 6], use of health services [7] and health expenditures [7, 8] in large and representative surveys of the general adult population. Considering that the concept, or latent variable, measured by the SRH item is still not entirely clear [9], evidence suggests that the SRH item captures a broad range of health dimensions [10]—physical, mental and functional health [11, 12]—and health behaviours [10, 12], and reflects an enduring rating of one’s health status [4]. The SRH item thus functions as an umbrella indicator of respondents’ general health status.

What the SRH item measures may be sensitive to the wording of the question and the response options. Indeed, the SRH item allows respondents to assess their health status according to their own definition of health. The SRH item is also a comprehensive assessment of health in general rather than an inquiry about specific symptoms, illnesses or dysfunctions. Various phrasings of the question and response options (hereafter, “forms”), however, have been used in population health surveys. For examples, common phrasings include “Would you say your health is…”, or “In general, how would you rate your health?” Common response options are “excellent/very good/good/fair/poor” or “very good, good, fair, bad, very bad”, but can also range from three to five options. For an illustration of the variability of forms in large-scale, national and international, survey infrastructures, see Table S1. As the SRH item is among the most frequently used health measures in population health surveys, it is important to determine if different forms are equivalent [13, 14], specifically in terms of the association of the SRH item with specific health measures (construct validity).

Numerous countries monitor the evolution of the health status of their general population with repeated cross-sectional surveys using the SRH item, but they often have changed its forms over time [15]. For example, the Swiss Health Survey (SHS) has conducted five repeated surveys between 1992 and 2012 and has modified the SRH item twice during this period (the phrasing of both the question and the response options), resulting in three different forms. In such context, it is important to determine if changes in the SRH form alter what the SRH item actually measures. Variations in the question [15] and in response options [13, 14, 16, 17] have been shown to alter the assessment of health; however, in the context of the SHS, both types of change (question and response options) are concurrent, a specific case which need to be evaluated. Here, we sought to examine (a) whether three forms of the SRH item in the SHS are equivalent in terms of construct validity and, if not, to determine (b) the form with the best construct validity and (c) the best coding scheme of response options to maximize equivalence across the three forms.

## Methods

### Study design

We used the SHS, a repeated, cross-sectional, nationally representative survey of residents 15 years and older living in Switzerland. The SHS surveyed participants in three languages (German, French and Italian), depending on their region of residency. The SHS was administered in 1992, 1997, 2002, 2007 and 2012 and the participation rates *(the number of participants having participated divided by the number of people invited to participate in the survey)* were 70.8%, 68.8%, 63.9%, 66.3% and 53.1%, respectively. For the analyses, we excluded respondents aged less than 18 (*N* = 2′780), and used data from the remaining 58,023 respondents who participated in waves 2002, 2007 and 2012. The SHS data are anonymous and available upon payment of fees. Ethical approval has been obtained by the Swiss Federal Statistical Office.

### Self-rated health forms

The three forms of the self-rated health item are detailed in appendix (Table S2).

### Coding schemes of response options

We used four coding schemes of response options (Table S3). First, we coded response options as a binary variable with an emphasis on positive options: “very good, good” versus “middle/moderate/ relatively good, poor, very poor”. This coding scheme was named “Dichotomised with positive focus”. Second, we coded response options as a binary variable with an emphasis on negative options: “very good, good, moderate/relatively good” versus “poor, very poor”. This coding scheme was named “Dichotomised with negative focus”. Third, we treated response options as linear. Fourth, we “linearised” response options with an alternative coding scheme, by recoding response options with ratings values: 1, 2, 3.7, 4.5 and 5, corresponding to an evenly spaced distance on a visual analogue scale [18]. Such transformation improves the interpretation of the mean values of SRH. This recoding has been developed for response options “excellent, very good, good, fair, poor” (three positive and two negative). This linearised coding scheme was applied to form 3, which was the only form to have adopted the “US form” for its responses, but not to forms 1 and 2 which response options were too different (two positive, one neutral, two negative). *These four schemes are the most frequently used treatment of response options in health research *[19–22].

### Health status variables

The SRH item captures a range of health dimensions [10]. Several health status variables were grouped into four dimensions: physical health, mental health, functional health [11, 23, 24] and health behaviours [10]. The first three dimensions mirror the WHO definition of health, which is “a state of complete physical, mental and social well-being” [25]. Physical health variables included body mass index (BMI), back pains, headaches, cardiac irregularities, chest pain, diarrhoea or constipation, fever and stomach pain or bloating. They also included chronic disease variables such as treatment for allergies, bronchitis, cancer or a tumour, hypertension, kidney stones, mental breakdown, myocardial infarction, stroke and diabetes in the 12 months that preceded the survey. Mental health variables included feeling unable to overcome barriers, loss of control, feeling overwhelmed with problems, feeling tired or exhausted or without energy, and problems with sleeping. Functional health variables included needing assistance to walk, to read and to hear. Health behaviour variables included smoking (yes, no), frequency of alcohol consumption (never, once a day and less, twice a day, three times a day), physical activity during free time, eating fruits daily and eating vegetables daily.

Response options of physical, mental, functional health and chronic disease variables were re-coded as present (1) vs. absent (0). Respondents with missing information were imputed as 0 (absence). BMI was defined following the Quetelet definition (kg/m^2^). All these health status variables were used as predictors of SRH.

### Covariates of self-rated health

We used the following known factors associated with SRH [19, 26–30]: age *(continuous)*, marital status (single, married, widowed, divorced and separated), number of children younger than 15 years living in the household (0, 1, 2, 3 and more), nationality (Swiss, other), education (primary, secondary, tertiary), household monthly income (≤ CHF 2000, CHF 2001–4000, CHF 4001–6000, > CHF 6000), employment status, urban vs. rural area of residency, linguistic region (German, French, Italian), use of medicine in the last 7 days (yes, no) and having friends or relatives to discuss personal issues (yes, no). Employment status had three categories: out of the labour force (including student, unemployed, retired and others), employed full time, and employed part time. Household income was weighted with the number of persons living the household and the number of children less than 15 years old.

### Statistical analyses

The three forms of SRH were used as dependent variables. Multivariable regression models were used to assess the contribution of health variables (thirty health status variables, representing thirty health predictors). *Linear regression was used when the coding schemes were continuous (linear and linearised) and logistic regression when coding schemes were binary (dichotomous with positive focus and with negative focus). All models were adjusted with covariates of SRH. Age was included as a continuous variable. In sensitivity analyse, age was used as category for stratification purpose (see section Sensitivity analyses)*. We computed the percentage of explained variances using the adjusted R squared for the linear coding scheme, the MacKelvey and Zavoina pseudo R squared for the dichotomous coding schemes, and reported these percentage of explained variances for the overall model (all health status variables) and by health dimensions (physical health, chronic diseases, mental health, functional health, health behaviours). Analyses were conducted overall and separately for women and men because gendered differences in the production of self-rated health assessments [31, 32].

The three SRH forms were administered at different periods (2002, 2007 and 2012); thus, differences across SRH forms may reflect “true” differences in health status of the general population. To limit the impact of these different periods, all models were adjusted with covariates known to be associated with SRH [19, 26–30]: age, marital status, number of children, nationality (Swiss or foreign), education, income, employment status, living in urban or rural area, linguistic regions, use of medicine in the last 7 days, and having friends or relatives to discuss personal issues.

When the events per variable (EPV) were smaller than 10, we did not estimate the model as they are known to produce incorrect estimates [33]. This occurred to the full model, including all covariates, and using the coding scheme “Dichotomised with negative focus” (out of 7300 patients, 320 had a value of very poor or poor and there were 45 predictors). However, we estimated the models using the coding scheme “Dichotomised with negative focus” for each health dimensions taken separately.

### Sensitivity analyses

First, we ran the same analyses on all waves of the SHS surveys, i.e. including waves 1992 and 1997 in which the SRH form was similar to form 1 (2002). Thus, the sample size for SRH form 1 increased to 20,873 men and 25,809 women. Second, we replicated the models stratifying by age groups: 18–35, 36–59 and 60 + . Health status ratings are age dependant [9, 34], as elderly people have been shown to be more optimistic [35–37]. Third, we replicated the models stratifying by education because evidence suggests that reliability of SRH may be lower among disadvantaged people [38, 39] and the meaning of rating may vary by education [34].

## Results

### Participant characteristics

**Fig. 1:**
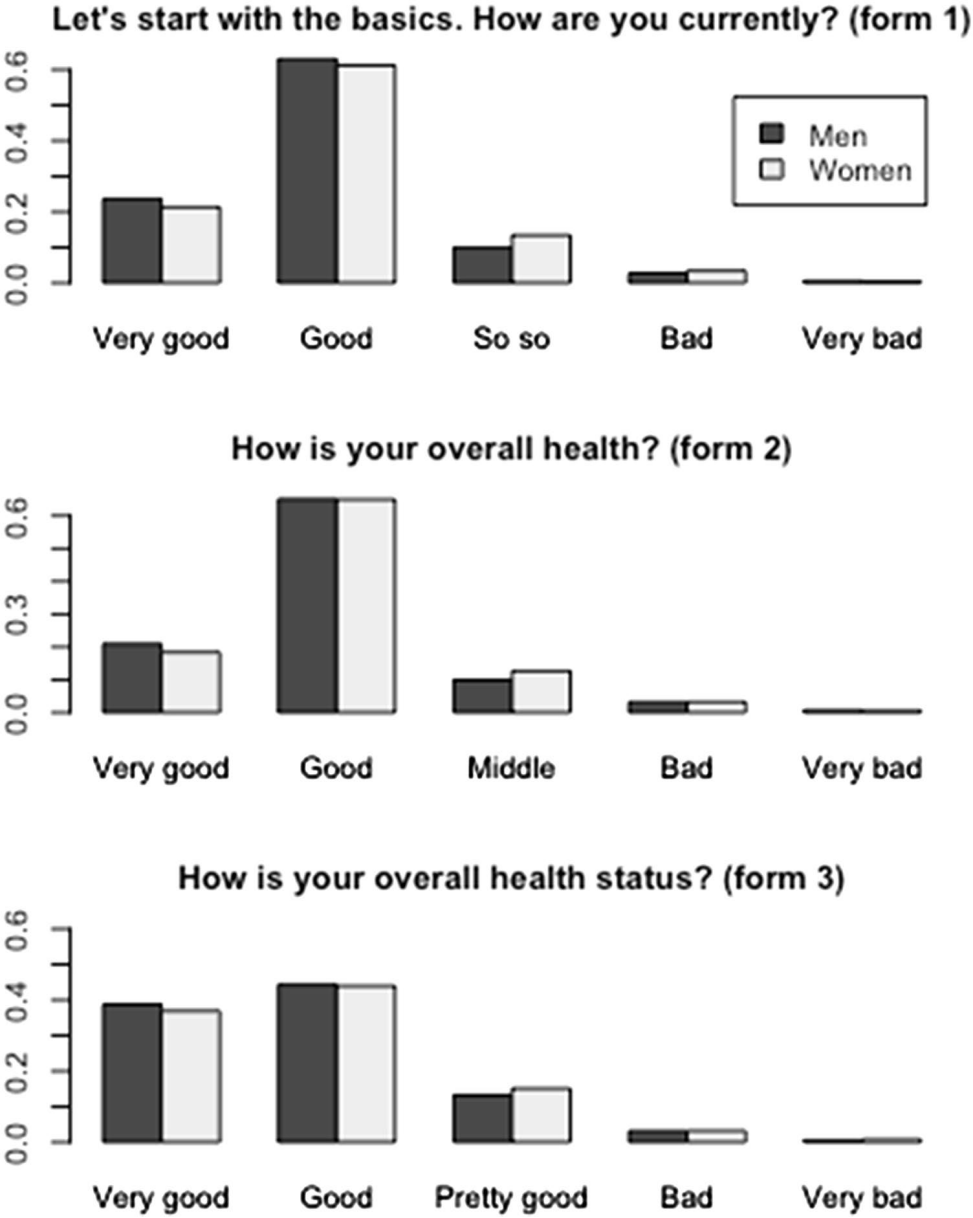
Distribution for each form of the SRH item. *SRH* self-rated health. Source: Swiss Health Survey

The sociodemographic characteristics of participants were different across the three forms of SRH (Table 1 and Appendix S4). Distributions of response options across the three forms of SRH were similar (Fig. 1) with the exceptions of respondents reporting “good” and “very good” health: the proportion of respondents reporting “good” health was lower in form 3 (44.1%) compared to forms 2 (65.0%) and 1 (58.6%). The proportion of respondents reporting “very good” health varied across forms 1, 2 and 3 (25.0%, 19.5% and 37.8%, respectively). Treated as a linear variable, the mean of the SRH item was 4.04 (95% Confidence Interval (CI) 4.04–4.05) in form 1, 3.99 (95% CI 3.98–4.01) in form 2 and 4.15 (95% CI 4.14–4.16) in form 3. Adjusted for participants’ characteristics (see list in Method section), the mean SRH depicted the same pattern across forms (4.06, 95% CI [4.06–4.06]; 4.02, 95% CI [4.02–4.03]; and 4.18, 95% CI [4.18–4.19], respectively). Standard deviation (SD) was higher in form 3 (0.83) compared to form 1 (0.75) and form 2 (0.71). These variations across SRH forms were similar for both men and women.

**Table 1:** Sample characteristics for three self-rated health forms, Swiss Health Survey

	SRH form 1* N (%)	SRH form 2* N (%)	SRH form 3* N (%)	*P*
Men				
Survey periods	2002	2007	2012	
Number of respondents	8620	8097	9859	
Age, mean (SD)	48.6 (16.9)	49.6 (17.3)	49.1 (17.8)	0.11
Marital status Single Married Widowed Divorced and separated	2288 (26.5) 5234 (60.7) 353 (4.1) 743 (8.6)	2234 (27.6) 4645 (57.4) 360 (4.4) 851 (10.5)	2898 (29.4) 5951 (60.4) 266 (2.7) 7336 (7.5)	< 0.001
Number of children younger than 15 living in the household 0 1 2 3 and more	6336 (73.5) 892 (10.3) 1042 (12.1) 350 (4.1)	6191 (76.5) 764 (9.4) 848 (10.5) 294 (3.6)	6964 (70.7) 1300 (13.2) 1175 (11.9) 413 (4.2)	< 0.001
Citizenship Swiss Other	7483 (86.8) 1137 (13.2)	6909 (85.4) 1184 (14.6)	8019 (81.3) 1840 (18.7)	< 0.001
Education Primary Secondary Tertiary	1121 (13.0) 5305 (61.6) 2182 (25.3)	758 (9.4) 4585 (56.7) 2750 (34.0)	1252 (12.8) 4953 (50.5) 3598 (36.7)	< 0.001
Household monthly income (CHF) Less than 3000 3000–6000 6001–9000 More than 9000	2231 (27.0) 4715 (57.0) 1029 (12.4) 292 (3.5)	2475 (31.4) 3940 (50.0) 1042 (13.2) 421 (5.3)	2063 (21.7) 5465 (57.6) 1401 (14.8) 564 (5.9)	< 0.001
Employment Out of labour force Employed full time Employed part time	2403 (28.2) 5503 (64.5) 630 (7.4)	2333 (29.1) 4903 (61.2) 778 (9.7)	2683 (27.5) 6071 (62.1) 1018 (10.4)	< 0.001
Urban area Urban Rural	6131 (71.1) 2489 (28.9)	5457 (67.4) 2640 (32.6)	7012 (71.1) 2847 (28.9)	< 0.001
Linguistic area German speaking French speaking Italian speaking	5901 (68.5) 2052 (23.8) 667 (7.7)	5001 (61.8) 2462 (30.4) 634 (7.8)	6614 (67.1) 2537 (25.7) 708 (7.2)	< 0.001
Use of medicine over the past 7 days	3348 (38.9)	3669 (45.4)	4542 (46.1)	< 0.001
Having friends or relatives to discuss personal issues	7520 (94.3)	7108 (93.4)	9040 (95.6)	< 0.001
Women				
Survey periods	2002	2007	2012	
Number of respondents	10,510	10,059	10,878	
Age, mean (SD)	50.6 (17.4)	51.6 (18.0)	49.9 (17.8)	0.005
Marital status Single Married Widow Divorced and separated	2322 (22.1) 5505 (52.4) 1450 (13.8) 1228 (11.7)	2323 (23.1) 4904 (48.8) 1521 (15.1) 1302 (13)	2625 (24.2) 5943 (54.7) 1009 (9.3) 1291 (11.9)	< 0.001
Citizenship Swiss Other	9331 (88.8) 1179 (11.2)	8884 (88.4) 1169 (11.6)	9205 (84.6) 1673 (15.4)	< 0.001
Education Primary Secondary Tertiary	2573 (24.5) 6889 (65.7) 1030 (9.8)	1793 (17.8) 6230 (62.0) 2027 (20.2)	1815 (16.8) 6558 (60.6) 2454 (22.7)	< 0.001
Number of children younger than 15 living in the household 0 1 2 3 and more	7941 (75.6) 1026 (9.8) 1134 (10.8) 408 (3.9)	7730 (76.8) 996 (9.9) 1018 (10.1) 315 (3.1)	7621 (70.1) 1499 (13.8) 1287 (11.8) 465 (4.3)	< 0.001
Income Less than 3000 3000–6000 6001–9000 More than 9000	3322 (34.1) 5541 (56.9) 712 (7.3) 162 (1.7)	3569 (37.9) 4885 (51.9) 763 (8.1) 203 (2.2)	2681 (26.6) 5988 (59.5) 1142 (11.3) 258 (2.6)	< 0.001
Employment				
Out of labour force	4952 (48.2)	4548 (46.6)	4174 (39.0)	< 0.001
Employed full time	2329 (22.7)	2180 (22.3)	2612 (24.4)	
Employed part time	2997 (29.2)	3042 (31.1)	3909 (36.5)	
Urban area				
Urban	7721 (73.5)	7011 (69.7)	7823 (71.9)	0.001
Rural	2788 (26.5)	3048 (30.3)	3055 (28.1)	
Linguistic area				
German speaking	6936 (66.0)	6109 (60.7)	7113 (65.4)	< 0.001
French speaking	2720 (25.9)	3084 (30.7)	2926 (26.9)	
Italian speaking	853 (8.1)	866 (8.6)	839 (7.7)	
Use of medicine over the past 7 days	5156 (49.1)	5617 (55.9)	5829 (53.6)	< 0.001
Having friends or relatives to discuss personal issues	9711 (95.4)	9249 (95.4)	10,075 (95.9)	0.09

*SRH* self-rated health

^*^Form 1: “Let’s start with the basics. How are you doing today?”, response options: very good, good, okay (moderate), bad, very bad;

Form 2: “How is your health in general?”, response options: very good, good, moderate, bad, very bad;

Form 3: “How is your health status in general? Would you say it is…”, response options: very good, good, relatively good, bad, very bad

### Construct validity equivalence between three forms of SRH

Thirty-four health status variables were used to predict the three SRH forms. The distribution of these variables across the three forms of SRH is reported in Table S5. Explained variances were estimated for three coding schemes (dichotomised with positive focus, dichotomised with negative focus and linear) in each form. Our fourth coding scheme (“linearised”) was specific to form 3 (Table 2). Results showed that the largest difference (across the SRH forms) of overall explained variances was 3.8% for linear coding and 4.2% for dichotomised with positive focus (dichotomised with negative focus was not estimated for the overall model). Across health dimensions, differences in explained variances across forms were less pronounced. We observed the lowest differences across forms for functional health (largest difference of explained variances less than 1% across coding schemes) and the highest for mental health (ranging 1.6–3.3% across coding schemes). Differences across forms for the other health dimensions fluctuated with coding schemes (chronic diseases 0.2–1.3%, physical health 0.4–1.9%, health behaviours 0.5–2.5%).

**Table 2:** Percentages of explained variance of three forms of self-rated health, overall and by health dimensions, by four coding schemes

	SRH form 1	SRH form 2	SRH form 3	Largest difference across three SRH forms
Dichotomised with positive focus coding: Very bad, bad, moderate *versus* good, very good				
Overall	12.7%	10.0%	14.2%	4.2%
Physical health	6.9%	5.5%	7.4%	1.9%
Chronic diseases	1.9%	1.7%	2.5%	0.8%
Mental health	6.7%	4.8%	7.6%	2.8%
Functional health	0.6%	0.4%	0.5%	0.2%
Health behaviours	2.2%	2.1%	2.6%	0.5%
Dichotomised with negative focus coding: Very bad, bad *versus* moderate, good, very good				
Overall^a^	**–**	**–**	**–**	**–**
Physical health	1.7%	1.3%	1.3%	0.4%
Chronic diseases	0.2%	0.0%	0.0%	0.2%
Mental health	2.1%	0.6%	2.2%	1.6%
Functional health	0.0%	0.0%	0.0%	0.0%
Health behaviours	3.0%	1.4%	3.9%	2.5%
Linear^b^ coding				
Overall	14.1%	12.9%	16.7%	3.8%
Physical health	8.0%	7.4%	9.3%	1.9%
Chronic diseases	2.8%	3.2%	4.1%	1.3%
Mental health	8.1%	6.7%	10.0%	3.3%
Functional health	1.3%	1.9%	1.2%	0.7%
Health behaviours	1.5%	1.5%	2.7%	1.2%
Linearised^c^ coding				
Overall	**–**	**–**	21.8%	**–**
Physical health	**–**	**–**	12.6%	**–**
Chronic diseases	**–**	**–**	4.9%	**–**
Mental health	**–**	**–**	13.3%	**–**
Functional health	**–**	**–**	2.1%	**–**
Health behaviours	**–**	**–**	3.1%	**–**

*SRH* self-rated health. Source: Swiss Health Survey

Adjusted R squared for linear coding scheme, MacKelvey and Zavoina pseudo R squared for dichotomous coding schemes. All models were adjusted for age, gender, marital status, number of children, nationality, education, income, employment status, urban vs. rural area, linguistic region, use of medicine in the last 7 days, having friends or relatives to discuss personal issues

^a^Model for “overall” when SRH was coded “dichotomised with negative focus” was not estimated because the ratio between the degrees of freedom and the sample size was lower than 10—see “Statistical analysis” section

^b^Linear = Response options coded 1–5

^c^Linearised = Response options coded “evenly” spaced: 1, 2, 3.7, 4.5, 5. The linearised coding scheme can be used with form 3 only—see “Method” section

Similar patterns were observed when stratifying the results between men and women (Table S6).

### SRH form with the best construct validity

Form 3 had the best overall explained variance in linear (16.7%) and dichotomised with positive focus (14.2%) coding schemes (Table 2). Across health dimensions, form 3 frequently explained more variance compared to forms 1 and 2, except when SRH was dichotomised with negative focus. Results were similar when stratifying the results between men and women (Table S6). In form 3, the linearised coding scheme explained more variance compared to the other coding schemes, overall and across health dimensions.

### Associations between SRH form 3 linearised and health status variables

*Using a multivariable linear regression model,* we further explored the associations between 34 health status variables and the SRH form having the best construct validity, i.e. form 3, coded “linearised” (Table *S7*). For both men and women, 26 health status variables were significantly associated with SRH. Among these, all associations were in the expected direction, except alcohol consumption: drinking twice a day was associated with better SRH compared to drinking once a day and less. Never drinking alcohol was associated in the expected direction, i.e. with higher SRH. Eight health status variables were not significantly associated with SRH, though six of these had coefficients in the expected direction.

### Maximising construct validity equivalence between the three forms of SRH

Considering the overall explained variance for men and women together, results showed that the linear scheme had the lowest variation between forms (standard deviation (SD) of overall explained variance = 1.94) compared to the dichotomised with positive focus scheme (Table 3). However, the best coding scheme was different for men and women: SD of overall explained variance was the lowest for the linear scheme for men (SD 1.96) and for the dichotomised with positive focus scheme for women (SD 1.72).

**Table 3:** Standard deviation of percentages of explained variance of self-rated health forms, by coding schemes, adjusted

	Coding schemes		
	Dichotomised with positive focus (very bad, bad, moderate *versus* good, very good)	Dichotomised with negative focus (very bad, bad *versus* moderate, good, very good)	Linear^a^
*All*	SD	SD	SD
Overall	2.13	–	1.94
Physical health	0.99	0.23	0.97
Chronic diseases	0.42	0.12	0.67
Mental health	1.43	0.90	1.66
Functional health	0.10	0.00	0.38
Health behaviours	0.26	1.27	0.69
*Men*			
Overall	2.04	–	1.96
Physical health	1.08	0.92	0.85
Chronic diseases	0.59	0.06	0.93
Mental health	1.35	0.64	1.30
Functional health	0.20	0.00	0.61
Health behaviours	0.53	1.79	0.85
*Women*			
Overall	1.72	–	1.95
Physical health	0.90	0.98	1.04
Chronic diseases	0.21	0.21	0.47
Mental health	1.36	1.01	1.96
Functional health	0.12	0.00	0.20
Health behaviours	0.40	0.86	0.59

*SD* standard deviation. Source: Swiss Health Survey

For age, gender, marital status, number of children, nationality, education, income, employment status, urban vs. rural area, linguistic region, use of medicine in the last 7 days, having friends or relatives to discuss personal issues

^a^Linear = Response options coded 1–5

For specific health dimensions, results were heterogeneous. In the full sample, SD of explained variance was the lowest in the dichotomised with negative focus scheme for all health dimensions except the dimension of health behaviours. A similar pattern was observed when stratifying the results between men and women, with the exception of physical health.

### Sensitivity analyses

First, analyses including respondents from waves 1992 and 1997 showed similar findings (data not shown), i.e. non-equivalence between forms, and form 3 with the best overall explained variance. Second, analyses stratified by age groups (18–35, 36–59 and 60 +) showed also similar findings with main analysis (data not shown). In terms of the coding scheme maximising the equivalence across forms, no clear pattern emerged from the results, with the exception that the dichotomised with positive focus scheme maximised equivalence among respondents aged 36–59. Twenty-three and 26 health status variables were significantly associated with SRH among respondents 18–35 and 36–59, respectively. These associations were in the excepted direction, with a few exceptions. Among respondents aged 60 + , 17 health status variables were significantly associated with SRH, and these associations were in the expected direction. Among the 17 health status variables not significantly associated with SRH, 11 of them had a coefficient in the expected direction and 6 in the unexpected direction. Third, analyses stratified by education groups showed similar findings with main analysis (non-equivalence between forms, form 3 with the best overall explained variance) for the three educational groups (data not shown). In terms of the coding scheme maximising the equivalence across forms, no clear pattern emerged from the results, except among respondents with secondary education where the dichotomised with positive focus scheme maximised equivalence. Twenty-nine and 25 health status variables were significantly associated with SRH among respondents with secondary and tertiary education, respectively, and these associations were in the excepted direction, with a few exceptions. Among respondents with primary education, 13 health status variables were significantly associated with SRH, and these associations were in the expected direction. Twenty-one health status variables were not associated with SRH; however, almost all of these were associated in the expected direction. Similarly to main the analysis, alcohol consumption was associated with SRH in the unexpected direction across all age and education groups.

## Discussion

The first objective of this study was to examine if the construct validity of three forms of the SRH item is equivalent. Differences in the percentages of the overall explained variance suggested that the three forms were not equivalent in their ability to capture respondents’ general health. The overall percentage of explained variance was 12.7% in form 1, 10.0% in form 2 and 14.2% in form 3. This difference was similar when using different coding schemes. When considering the association between different health dimensions and the SRH item, however, the lack of equivalence was less pronounced. Functional health was the dimension with the best, almost strict, equivalence across forms: for this dimension, differences across the forms were systematically less than 1%, regardless of coding schemes. In other words, respondents were not influenced by the form of the SRH when assessing the functional aspect of their health, like their autonomy (walking, reading and hearing). Conversely, mental health was the dimension with the worst equivalence across forms: differences of explained variances across forms were systematically above 1% across coding schemes, with a largest difference of 3.8% for linear coding. In other words, change in SRH forms may influence the way respondents are assessing the mental aspect of their general health, like their sleep quality, sense of control, feeling of tiredness, etc.

The second objective of this study was to determine which form had the best construct validity. Form 3 (“How is your health status in general? Would you say it is…”) had the best construct validity compared to forms 1 and 2, since it had the highest percentage of explained variance. This result holds when using two different coding schemes (linear and dichotomised with positive focus) and when looking across health dimensions of SRH items. This finding is expected because form 3 has a clearer, more focused, question about health in general compared to form 1 (“How are you doing today?”). Form 1 relates to a general inquiry about how life is going, or someone’s health, or someone’s actual emotional makeup, or someone’s satisfaction with life. Form 2 (“How is your health in general?”) is the closest to the frequently used form in national surveys and epidemiological studies (form 9 in Table S1). Two differences characterise form 2 and form 3. First, form 3 focuses on “health status” while form 2 focuses on “health” alone. Second, form 3 contrasts three positive response options against two negative options, also called the “US version”, while form 2 is more balanced by contrasting two positive, one neutral, and two negative options, also called the “WHO version” [14]. In high-income countries, where prevalence of good general health is high, giving respondents three shades of positive ratings may be more appropriate to capture inter-individual variability, in contrary to middle- and low-income countries, where prevalence of poor health suggests to prefer the WHO version [2].

A striking finding was the good performance of form 1 (“Let’s start with the basics. How are you doing today?”). At first glance, this form potentially steers away from a question assessing health status but, in our results, it performed better compared to form 2 (“How is your health in general?”), one of the most commonly used form in health research (see Table S1). This finding supports the hypothesis that form 1 of the SRH item may be an indicator of general well-being [40, 41].

We also showed that transforming the numerical distribution of response options into a way that linearised the intervals between the five options [18] allowed to increase the explained variance. This transformation only applied to form 3, which sets up three positive statements against two negatives. Such transformation is simple, improves statistical analysis, allows keeping respondents’ answer in their original response options (instead of dichotomising response options), and improves the interpretation of mean values of SRH in populations groups [18].

The third objective of this study was to determine the coding scheme maximizing the equivalence across the three forms, to improve comparison across SHS waves. Based on the results, we advise using the linear coding scheme which had the lowest variation across forms in the proportion of explained variance in SRH by 34 health variables. In other words, studies wanting to use the SRH item across different waves (trend studies) [42] should treat this item as a continuous variable instead of a dichotomous one. However, this recommendation may not apply to studies examining one gender only: in a study of women, the dichotomous coding scheme with negative focus (very bad, bad, moderate *vs*. good, very good) is recommended to maximise the equivalence between forms of the SRH.

The above findings are robust in the sense that we used a large sample size, the quality of the data from the SHS was high, and the explained variance was adjusted for demographics, socio-economic and family factors associated with SRH [19, 26–30]. Our main findings were also confirmed by two sensitivity analyses, one by age groups and one by educational groups. However, sensitivity analyses did not confirm the finding that using the linear coding scheme should maximise the equivalence between forms of the SRH: it was not true for the 36–59 years old and the secondary educated, for whom the best coding scheme was the dichotomous with positive focus; and for maximising the equivalence across SRH forms, using the dichotomous coding scheme with negative focus is recommended for studies on women. Similarly, sensitivity analyses suggested that construct validity of the best form (form 3) was a bit lower among vulnerable or disadvantaged people, like the 60 + and those with primary education, a result in line with other studies [34, 43]. At the same time, *several limitations of our study have to be considered. First,* our findings suffer from lack of *time* synchronicity between waves *(so between the different SRH forms). However, all models were adjusted with numerous sociodemographic and socioeconomic covariates to minimise this lack of temporal synchronicity. Second, the study has been conducted in one single country, Switzerland, thus its generalisability is subject to caution. However, studies have suggested construct validity of SRH was similar across very different countries* [2]*. Third, almost all SRH’s covariates, be they health factors, health behaviours, health problems, demographic characteristics, socioeconomic status, *etc*., were significantly different across waves. In the Swiss health interview data, the 3 forms of SRH have been used at different waves, so waves and SRH forms are confounded. Because we do not have different SRH forms measured synchronously (during the same year), it is impossible to disentangle the effect of time change from the effect of different SRH forms. Finally, we used adjusted coefficients of determination (R squared when SRH was treated as a linear variable, MacKelvey and Zavoina pseudo R squared when SRH was treated dichotomous) to assess relative construct validity. However, the comparison does not include a p-value to determine which scheme was significantly more informative than another, and thus our comparison is more qualitative than inferential.*

In conclusion, the three forms of the SRH item in the SHS were not equivalent in terms of their relationships with other health measures. Institutions conducting repeated population health and demographic surveys and using this item should strive to keep the SRH item similar across waves. The form with the best construct validity was “How is your health status in general?”. For studies aiming at examining the evolution of SRH over time, the linear coding scheme was the best option to maximise equivalence between SRH forms for the overall population. However, other coding performed better for subgroups and thus different coding options should systematically be investigated.

## Electronic supplementary material

Below is the link to the electronic supplementary material.

Supplementary file1 (DOCX 48 kb)

## Data Availability

This study used the data from the five waves of the Swiss Health Survey. Data are available for fee (1500 Swiss Francs, plus 8.0% tax) and users must request permission from the Swiss Federal Statistical Office (sgb12@bfs.admin.ch). Data must be destroyed after five years.
