# Supplementary material for: Does the single-item self-rated health measure the same thing across different wordings? Construct validity study
Source: Qual Life Res. 2020 May 20;29(9):2593–604. doi: 10.1007/s11136-020-02533-2 (PMC7434800; doi:10.1007/s11136-020-02533-2)
Supplement: Supplementary file 1 — Supplementary file1 (DOCX 48 kb) [file 11136_2020_2533_MOESM1_ESM.docx]

**On-line Supplementary Appendices**

**Table S1 Forms of the self-rated health item in large-scale, national and international research survey infrastructures**

**Table S2 Forms of the self-rated health item in the Swiss Health Survey, with original wordings in German, French and Italian**

**Table S3 Coding schemes of response options of the self-rated health item**

**Appendix S4 Participants’ characteristics**

**Table S5 Distribution of health characteristics across three forms of self-rated health**

**Table S6 Percentages of explained variance of three forms of self-rated health, overall and with health dimensions, by four coding schemes, among men and women**

**Table S7 Association of self-rated health form 3, linearised coding scheme, with 30 health status variables**

**Table S1 Forms of the self-rated health item in large-scale, national and international, survey infrastructures**

|  | **Questions** | **Response options** | | **Examples of survey using the form** |
| --- | --- | --- | --- | --- |
|  |  | **Number** | **Labels** |  |
| 1 | In general, would you say your health is… | 5 | excellent, very good, good, fair, poor | Canadian National Population Health Survey;  New Zealand Survey of Families, Income and Employment |
| 2 | In general, would you say your health is… | 5 | very good, good, satisfactory, poor, very poor | Aging, Status, and the Sense of Control (US) |
| 3 | In general, how would you rate your health today? | 5 | very good, good, moderate, bad, very bad | World Health Survey (World Health Organisation) |
| 4 | In general, would you say your physical health is... | 5 | excellent, very good, good, fair, poor | Midlife in the United States |
| 5 | Would you say your health in general is... | 5 | poor, fair, good, very good, excellent | Panel Study of Income Dynamics (US);  National Health Epidemiologic Follow-up Survey (US) |
| 6 | Would you say your own health, in general, is… | 4 | excellent, good, fair, poor | General Social Survey (US) |
| 7 | Over the last 12 months would you say your health has on the whole been… | 3 | good, fairly good, not good | General Household Survey of Great Britain (until 2007) |
| 8 | Please think back over the last 12 months about how your health has been. Compared to people of your own age, would you say that your health has on the whole been... | 5 | excellent, good, fair, poor, very poor | [British Household Panel Survey](https://www.iser.essex.ac.uk/bhps) |
| 9 | How is your health in general? Is it... | 5 | very good, good, fair, bad, very bad | European Health Interview Survey;  Health Survey for England;  General Household Survey of Great Britain (since 2008)  Health Interview Survey (Belgium);  European Community Household Panel;  European Social Survey |
| 10 | How would you describe your current health: | 5 | very good, good, satisfactory, poor, bad | German Socio-Economic Panel |
| 11 | How would you describe your health compared to that of other people your age? | 4 | excellent, good, fair, poor | China Health and Nutrition Survey |
| 12 | How would you rate your health in general? | 4 | excellent, good, fair, poor | Australian longitudinal panel study |
| 13 | How would you rate your health at the present time? Would you say it is...? | 5 | excellent, very good, good, fair, poor | American's Changing Lives |
| 14 | How do you assess your general health? | 5 | very good, good, fair, poor, very poor | Swedish Survey of Living Conditions |
| 15 | Compared to other people your age, how would you describe your health? | 5 | very poor, poor, fair, good, excellent | National Survey of Families and Households (US) |
| 16 | We are now going to talk about various aspects of your health. How do you feel right now? | 5 | very well, well, so so, not very well, not well at all | Swiss Household Panel |

**Table S2 Forms of the self-rated health item in the Swiss Health Survey, with original wordings in German, French and Italian**

|  | **Language** | **SRH form 1** | **SRH form 2** | **SRH form 3** |
| --- | --- | --- | --- | --- |
| ***Question wording*** | German | Zuerst gerade das Nächstliegende: Wie geht es Ihnen zur Zeit gesundheitlich? | Wie ist Ihre Gesundheit im Allgemeinen? | Wie ist Ihr Gesundheitszustand im Allgemeinen? Ist er… |
|  | French | Commençons par l'essentiel : Comment allez-vous en ce moment ? | Comment est votre santé en général ? | Comment est votre état de santé en général ? Est-il… |
|  | Italiano | Cominciamo dall'essenziale: Come sta di salute in questo momento? | Come è la Sua salute in generale? | Come va in generale la sua salute? |
|  | *English translation* | *Let’s start with the basics. How are you* *doing today?* | *How is your health in general?* | *How is your health status in general? Would you say it is…* |
| ***Response options*** | German | Sehr gut  Gut  Mittelmässig  Schlecht  Sehr schlecht | Sehr gut  Gut  Mittelmässig  Schlecht  Sehr schlecht | Sehr gut  Gut  Mittelmässig  Schlecht  Sehr schlecht |
|  | French | Très bien  Bien  Comme ci, comme ça (moyen)  Mal  Très mal | Très bonne  Bonne  Moyenne  Mauvaise  Très mauvaise | Très bon  Bon  Assez bon  Mauvais  Très mauvais |
|  | Italiano | Molto bene  Bene  Mediamente  Non molto bene  Male | Molto buona  Buona  Normale  Scadente  Molto scadente | Molto bene  Bene  Discretamente  Male  Molto male |
|  | *English translation* | *Very good*  *Good*  *Okay (Moderate)*  *Bad*  *Very bad* | *Very good*  *Good*  *Moderate*  *Bad*  *Very bad* | *Very good*  *Good*  *Relatively good*  *Bad*  *Very bad* |

In form 1, the question was worded “Let’s start with the basics. How are you doing today?” and response options were very good, good, okay (moderate), bad, very bad. This form was administered in waves 1992, 1997, and 2002. For the main analyses, we used wave 2002. In sensitivity analyses, we pooled waves 1992, 1997, and 2002 together to check if results changed. In form 2, self-rated health was worded “How is your health in general?”, with response options including “very good, good, moderate, bad, very bad”. This form was administered in 2007. In form 3, the question was “How is your health status in general? Would you say it is...”, and the answers were “very good, good, relatively good, bad, very bad”. This form was administered in 2012. The framing of the question was thus different between form 1 versus forms 2 and 3. In form 1, the question asked respondents to rate how their current state is (“how are you doing today?”). In forms 2 and 3, the question asked respondents to rate their “health in general” (form 2) and their “health status in general” (form 3) (Table S2). Thus, form 1 addressed respondents’ well-being status, while forms 2 and 3 addressed the concept of respondents’ health. Another important difference is that form 2 mentions “health”, while form 3 mentions “health status”. Response options were different across forms due to the wording of the question. In form 1, respondents rated their status with adverbs of manner. In form 2 and 3, respondents rated their status with adjectives. Also, forms 1 and 2 have two positive and two negative options separated by a neutral one, when form 3 has three shades of positive against two negative options.

**Table S3 Coding schemes of response options of the self-rated health item**

|  | **SRH form 1** | **SRH form 2** | **SRH form 3** |
| --- | --- | --- | --- |
| Linear | 1. very bad  2. bad  3. okay (Moderate)  4. good  5. very good | 1. very bad  2. bad  3. moderate  4. good  5. very good | 1. very bad  2. bad  3. relatively good  4. good  5. very good |
| Linearised* | - | - | 1. very bad  2. bad  3.7. relatively good  4.5. good  5. very good |
| Dichotomised with positive focus | very bad, bad, okay (Moderate)  vs.  good, very good | very bad, bad, moderate  vs.  good, very good | very bad, bad, relatively good  vs.  good, very good |
| Dichotomised with negative focus | very bad, bad  vs.  okay (Moderate), good, very good | very bad, bad  vs.  moderate, good, very good | very bad, bad  vs.  relatively good, good, very good |

Abbreviation: SRH, self-rated health; Vs., versus

Source: Swiss Health Survey

* alternative coding scheme recoding response options with evenly spaced ratings values [[18](#_ENREF_18)]. This alternative coding is applicable to form 3 only.

**Appendix S4 Participants’ characteristics**

Over the period 2002-2012, societal changes occurred in Switzerland. Among men, we observed a decline in proportions of men with secondary education, income lower than 3000 Swiss francs, and Swiss citizenship. We also noted an increase in proportions of men with tertiary education, income above 6000 Swiss francs (6001-9000, >9000), working part time, living in households without children under 15, and having used medicine over the past 7 days. Other characteristics were either stable or fluctuant over time. Among women, we observed a decline in proportions of women with primary and secondary education, income lower than 3000 Swiss francs, and out of the labour force. We noted an increase in proportions of women with tertiary education, income above 3000 Swiss francs (3001-6000, 6001-9000, and >9000), working part time, and having friends or relatives to discuss personal issues. As was the case for men, other characteristics were either stable or fluctuant.

**Table S5 Distribution of health characteristics across three forms of self-rated health**

| **Men** | **SRH dichotomised**^‡^**, form 1*** | **SRH dichotomised**^‡^**, form 2*** | **SRH dichotomised**^‡^**, form 3*** |  |
| --- | --- | --- | --- | --- |
| Survey periods | 2002 | 2007 | 2012 |  |
|  | **N (%)** | **N (%)** | **N (%)** | ***p*** |
| **Physical health** | | | | |
| BMI  Underweight  Normal weight  Overweight  Obese | 320 (3.7%)  4125 (48.2%)  3351 (39.1%)  767 (9.0%) | 54 (0.7%)  3994 (49.8%)  3217 (40.1%)  752 (9.4%) | 80 (0.8%)  4574 (46.7%)  4020 (41.0%)  1130 (11.5%) | <0.001 |
| Back pain | 733 (8.5%) | 707 (8.7%) | 526 (5.3%) | <0.001 |
| Headaches | 429 (5.0%) | 391 (4.8%) | 279 (2.8%) | <0.001 |
| Cardiac irregularities | 101 (1.2%) | 124 (1.5%) | 95 (1.0%) | 0.002 |
| Chest pain | 113 (1.3%) | 93 (1.1%) | 74 (0.8%) | <0.001 |
| Diarrhoea, constipation | 215 (2.5%) | 216 (2.7%) | 189 (1.9%) | 0.002 |
| Fever | 87 (1.0%) | 85 (1.0%) | 64 (0.6%) | 0.006 |
| Stomach pain, bloating | 182 (2.1%) | 217 (2.7%) | 169 (1.7%) | <0.001 |
| **Chronic diseases** | | | | |
| Treatment for allergies (in the past 12 m.) | 603 (7.0%) | 306 (3.8%) | 436 (4.4%) | <0.001 |
| Treatment for bronchitis (in the past 12 m.) | 239 (2.8%) | 111 (1.4%) | 115 (1.2%) | <0.001 |
| Treatment for cancer or a tumour (in the past 12 m.) | 174 (2.0%) | 83 (1.0%) | 131 (1.3%) | <0.001 |
| Treatment for hypertension (in the past 12 m.) | 1075 (12.5%) | 880 (10.9%) | 1276 (12.9%) | <0.001 |
| Treatment for kidney stones (in the past 12 m.) | 181 (2.1%) | 73 (0.9%) | 109 (1.1%) | <0.001 |
| Treatment for mental breakdown (in the past 12 m.) | 282 (12.3%) | 223 (9.7%) | 264 (11.5%) | 0.04 |
| Treatment for myocardial infarction (in the past 12 m.) | 242 (2.8%) | 113 (1.4%) | 126 (1.3%) | <0.001 |
| Treatment for stroke (in the past 12 m.) | 77 (0.9%) | 44 (0.5%) | 41 (0.4%) | <0.001 |
| Diabetes | 389 (4.5%) | 482 (6.0%) | 593 (6.0%) | <0.001 |
| **Mental health** | | | | |
| Feeling unable to overcome barriers | 1025 (11.9%) | 916 (11.3%) | 1195 (12.1%) | 0.24 |
| Loss of control | 589 (6.8%) | 579 (7.2%) | 782 (7.9%) | 0.01 |
| Feeling overwhelmed with problems | 757 (8.8%) | 657 (8.1%) | 880 (8.9%) | 0.13 |
| Feeling tired, exhausted, without energy | 457 (5.3%) | 456 (5.6%) | 407 (4.1%) | <0.001 |
| Problems with sleeping | 508 (5.9%) | 548 (6.8%) | 463 (4.7%) | <0.001 |
| **Functional health** | | | | |
| Assistance to walk | 64 (0.7%) | 81 (1.0%) | 70 (0.7%) | 0.07 |
| Assistance to read | 119 (1.4%) | 94 (1.2%) | 134 (1.4%) | 0.38 |
| Assistance to hear | 95 (1.1%) | 130 (1.6%) | 107 (1.1%) | 0.002 |
| **Health behaviours** | | | | |
| Smoking | 3059 (35.5%) | 2588 (32.0%) | 3075 (31.2%) | <0.001 |
| Alcohol consumption frequency  Never  Once a day and less  Twice a day  Three times a day | 1045 (12.1%)  6972 (80.9%)  514 (6.0%)  89 (1.0%) | 781 (9.6%)  6844 (84.5%)  404 (5.0%)  68 (0.8%) | 968 (9.8%)  8404 (85.2%)  409 (4.1%)  78 (0.8%) | <0.001 |
| Physical activity in free time | 5193 (60.2%) | 5192 (64.1%) | 6497 (65.9%) | <0.001 |
| Eating fruits daily | 7955 (92.3%) | 5896 (72.8%) | 9443 (95.8%) | <0.001 |
| Eating vegetables daily | 8531 (99.0%) | 6272 (77.5%) | 9760 (99.0%) | <0.001 |
| **Women** | **SRH dichotomised**^‡^**, form 1*** | **SRH dichotomised**^‡^**, form 2*** | **SRH dichotomised**^‡^**, form 3*** |  |
| Survey periods | 2002 | 2007 | 2012 |  |
|  | **N (%)** | **N (%)** | **N (%)** | ***p*** |
| **Physical health** | | | | |
| BMI  Underweight  Normal weight  Overweight  Obese | 1917 (18.5%)  5204 (50.3%)  2398 (23.2%)  826 (8.0%) | 560 (5.7%)  6218 (63.1%)  2248 (22.8%)  836 (8.5%) | 631 (5.9%)  6601 (61.5%)  2468 (23.0%)  1033 (9.6%) | <0.001 |
| Back pain | 1420 (13.5%) | 1280 (12.7%) | 998 (9.2%) | <0.001 |
| Headaches | 1039 (9.9%) | 905 (9.0%) | 792 (7.3%) | <0.001 |
| Cardiac irregularities | 188 (1.8%) | 231 (2.3%) | 160 (1.5%) | <0.001 |
| Chest pain | 144 (1.4%) | 174 (1.7%) | 130 (1.2%) | 0.004 |
| Diarrhoea, constipation | 499 (4.7%) | 529 (5.3%) | 440 (4.0%) | <0.001 |
| Fever | 110 (1.0%) | 123 (1.2%) | 77 (0.7%) | 0.001 |
| Stomach pain, bloating | 450 (4.3%) | 473 (4.7%) | 404 (3.7%) | 0.002 |
| **Chronic diseases** | | | | |
| Treatment for allergies (in the past 12 m.) | 837 (8.0%) | 519 (5.2%) | 633 (5.8%) | <0.001 |
| Treatment for bronchitis (in the past 12 m.) | 317 (3.0%) | 160 (1.6%) | 182 (1.7%) | <0.001 |
| Treatment for cancer or a tumour (in the past 12 m.) | 269 (2.6%) | 161 (1.6%) | 227 (2.1%) | <0.001 |
| Treatment for hypertension (in the past 12 m.) | 1373 (13.1%) | 1050 (10.4%) | 1193 (11.0%) | <0.001 |
| Treatment for kidney stones (in the past 12 m.) | 164 (1.6%) | 69 (0.7%) | 91 (0.8%) | <0.001 |
| Treatment for mental breakdown (in the past 12 m.) | 586 (25.6%) | 441 (19.3%) | 492 (21.5%) | <0.001 |
| Treatment for myocardial infarction (in the past 12 m.) | 127 (1.2%) | 70 (0.7%) | 50 (0.5%) | <0.001 |
| Treatment for stroke (in the past 12 m.) | 62 (0.6%) | 34 (0.3%) | 32 (0.3%) | 0.001 |
| Diabetes | 429 (4.1%) | 485 (4.8%) | 453 (4.2%) | 0.02 |
| **Mental health** | | | | |
| Feeling unable to overcome barriers | 1507 (14.3%) | 1286 (12.8%) | 1567 (14.4%) | <0.001 |
| Loss of control | 779 (7.4%) | 734 (7.3%) | 877 (8.1%) | 0.08 |
| Feeling overwhelmed with problems | 1187 (11.3%) | 1054 (10.5%) | 1214 (11.2%) | 0.13 |
| Feeling tired, exhausted, without energy | 873 (8.3%) | 932 (9.3%) | 940 (8.6%) | 0.048 |
| Problems with sleeping | 1049 (10.0%) | 1220 (12.1%) | 965 (8.9%) | <0.001 |
| **Functional health** | | | | |
| Assistance to walk | 96 (0.9%) | 151 (1.5%) | 114 (1.0%) | <0.001 |
| Assistance to read | 159 (1.5%) | 173 (1.7%) | 160 (1.5%) | 0.30 |
| Assistance to hear | 69 (0.7%) | 120 (1.2%) | 83 (0.8%) | <0.001 |
| **Health behaviours** | | | | |
| Smoking | 2760 (26.3%) | 2374 (23.6%) | 2647 (24.3%) | <0.001 |
| Alcohol consumption frequency  Never  Once a day and less  Twice a day  Three times a day | 3131 (29.8%)  7181 (68.3%)  167 (1.6%)  31 (0.3%) | 2184 (21.7%)  7717 (76.7%)  149 (1.5%)  9 (0.1%) | 2268 (20.8%)  8444 (77.6%)  150 (1.4%)  16 (0.1%) | <0.001 |
| Physical activity in free time | 5698 (54.2%) | 5812 (57.8%) | 6205 (57.0%) | <0.001 |
| Eating fruits daily | 10035 (95.5%) | 8625 (85.7%) | 10568 (97.2%) | <0.001 |
| Eating vegetables daily | 10452 (99.4%) | 8849 (87.8%) | 10821 (99.5%) | <0.001 |

Abbreviation: SRH, self-rated health

Source: Swiss Health Survey

*Note:

Form 1: “Let’s start with the basics. How are you currently?”, response options: very good, good, okay (moderate), bad, very bad;

Form 2: “How is your health in general?”, response options: very good, good, moderate, bad, very bad;

Form 3: “how is your health status in general? Would you say it is…”, response options: very good, good, relatively good, bad, very bad.

**Table S6 Percentages of explained variance^a^ of three forms of self-rated health, overall and with health dimensions, by four coding schemes, among men and women.**

|  | Men | | | | Women | | | |
| --- | --- | --- | --- | --- | --- | --- | --- | --- |
|  | SRH Form 1 | SRH Form 2 | SRH Form 3 | Largest difference across forms | SRH Form 1 | SRH Form 2 | SRH Form 3 | Largest difference across forms |
| ***Dichotomised with positive focus coding: very bad, bad, moderate versus good, very good*** |  |  |  |  |  |  |  |  |
| **Overall** | **12.0%** | **9.3%** | **13.3%** | **4.0%** | **14.9%** | **12.2%** | **15.4%** | **3.2%** |
| Physical health | 6.6% | 4.5% | 6.0% | 2.1% | 8.0% | 7.0% | 8.8% | 1.8% |
| Chronic diseases | 1.3% | 1.5% | 2.4% | 1.1% | 2.4% | 2.3% | 2.7% | 0.4% |
| Mental health | 5.5% | 4.0% | 6.7% | 2.7% | 7.8% | 5.8% | 8.4% | 2.6% |
| Functional health | 0.4% | 0.2% | 0.6% | 0.4% | 0.7% | 0.5% | 0.5% | 0.2% |
| Health behaviours | 2.7% | 1.7% | 2.5% | 1.0% | 2.1% | 2.6% | 2.9% | 0.8% |
| ***Dichotomised with negative focus coding: very bad, bad versus moderate, good, very good*** |  |  |  |  |  |  |  |  |
| **Overall^b^** | **-** | **-** | **-** | **-** | **-** | **-** | **-** | **-** |
| Physical health | 0.6% | 0.0% | 1.8% | 1.8% | 3.1% | 3.6% | 1.7% | 1.9% |
| Chronic diseases | 0.0% | 0.0% | 0.1% | 0.1% | 0.5% | 0.6% | 0.2% | 0.4% |
| Mental health | 1.2% | 0.1% | 1.2% | 1.1% | 3.4% | 1.7% | 3.5% | 1.8% |
| Functional health | 0.0% | 0.0% | 0.0% | 0.0% | 0.0% | 0.0% | 0.0% | 0.0% |
| Health behaviours | 3.7% | 0.5% | 3.5% | **3.2%** | 2.6% | 3.7% | 4.3% | **1.7%** |
| ***Linear^b^ coding*** |  |  |  |  |  |  |  |  |
| **Overall** | **12.6%** | **12.6%** | **16.0%** | **3.4%** | **15.5%** | **13.5%** | **17.4%** | **3.9%** |
| Physical health | 7.1% | 6.3% | 8.0% | 1.7% | 8.9% | 8.4% | 10.4% | 2.0% |
| Chronic diseases | 2.5% | 3.0% | 4.3% | 1.8% | 3.1% | 3.3% | 4.0% | 0.9% |
| Mental health | 6.9% | 6.8% | 9.1% | 2.3% | 9.0% | 6.7% | 10.6% | 3.9% |
| Functional health | 1.0% | 2.1% | 1.1% | 1.1% | 1.5% | 1.7% | 1.3% | 0.4% |
| Health behaviours | 1.5% | 1.2% | 2.8% | 1.6% | 1.5% | 1.7% | 2.6% | 1.1% |
| ***Linearised^d^ coding*** |  |  |  |  |  |  |  |  |
| **Overall** | **-** | **-** | **21.8%** | **-** | **-** | **-** | **22.1%** | **-** |
| Physical health | - | - | 11.5% | - | - | - | 13.7% | - |
| Chronic diseases | - | - | 5.3% | - | - | - | 4.5% | - |
| Mental health | - | - | 13.1% | - | - | - | 13.5% | - |
| Functional health | - | - | 1.8% | - | - | - | 2.4% | - |
| Health behaviours | - | - | 3.1% | - | - | - | 3.1% | - |

Source: Swiss Health Survey

^a^adjusted R squared for linear coding scheme, MacKelvey and Zavoina pseudo R squared for dichotomous coding schemes. Models were adjusted for age, marital status, number of children, nationality, education, income, employment status, urban vs. rural area, linguistic region, use of medicine in the last 7 days, having friends or relatives to discuss personal issues

^b^ Model for “overall” was not estimated because the ratio between the degrees of freedom and the sample size was lower than 10 – see Statistical Analysis section.

^c^ Linear = Response options coded 1 to 5

^d^ Linearised = Response options coded “evenly” spaced: 1, 2, 3.7, 4.5, 5. The linearised coding scheme can be used with form 3 only.

**Table S7 Association of self-rated health form 3*, linearised coding scheme, with 30 health status variables.**

|  | Men | | Women | |
| --- | --- | --- | --- | --- |
|  | Coef | 95% CI | Coef | 95% CI |
| **Physical health** |  |  |  |  |
| BMI (ref. normal weight) |  |  |  |  |
| Underweight | -0.04 | -0.16 , 0.09 | -0.02 | -0.07 , 0.02 |
| Overweight | -0.05 | -0.07 , -0.02 | -0.06 | -0.08 , -0.03 |
| Obese | -0.09 | -0.12 , -0.05 | -0.13 | -0.16 , -0.09 |
| Back pain | -0.46 | -0.51 , -0.41 | -0.40 | -0.44 , -0.36 |
| Headaches | -0.14 | -0.20 , -0.07 | -0.13 | -0.17 , -0.09 |
| Cardiac irregularities | -0.50 | -0.62 , -0.39 | -0.16 | -0.25 , -0.06 |
| Chest pain | -0.34 | -0.48 , -0.20 | -0.23 | -0.34 , -0.13 |
| Diarrhoea, constipation | -0.18 | -0.26 , -0.09 | -0.15 | -0.21 , -0.09 |
| Fever | -0.10 | -0.23 , 0.04 | 0.10 | -0.03 , 0.22 |
| Stomach pain, bloating | -0.06 | -0.15 , 0.03 | -0.17 | -0.24 , -0.11 |
| **Chronic diseases** |  |  |  |  |
| Treatment for allergies (past 12 m.) | -0.00 | -0.05 , 0.05 | -0.04 | -0.09 , 0.00 |
| Treatment for bronchitis (past 12 m.) | -0.18 | -0.27 , -0.08 | -0.16 | -0.25 , -0.08 |
| Treatment for cancer or a tumour (past 12 m.) | -0.40 | -0.49 , -0.31 | -0.25 | -0.32 , -0.18 |
| Treatment for hypertension (past 12 m.) | -0.03 | -0.06 , 0.00 | -0.01 | -0.05 , 0.02 |
| Treatment for kidney stones (past 12 m.) | -0.13 | -0.22 , -0.03 | -0.07 | -0.19 , 0.04 |
| Treatment for mental breakdown (past 12 m.) | -0.30 | -0.36 , -0.23 | -0.16 | -0.22 , -0.11 |
| Treatment for myocardial infarction (past 12 m.) | -0.22 | -0.31 , -0.13 | -0.19 | -0.35 , -0.04 |
| Treatment for stroke (past 12 m.) | -0.17 | -0.32 , -0.01 | -0.16 | -0.34 , 0.03 |
| Diabetes | -0.20 | -0.25 , -0.15 | -0.18 | -0.24 , -0.13 |
| **Mental health** |  |  |  |  |
| Feeling unable to overcome barriers | -0.08 | -0.12 , -0.04 | -0.06 | -0.10 , -0.03 |
| Loss of control | -0.06 | -0.10 , -0.02 | -0.04 | -0.09 , -0.00 |
| Feeling overwhelmed with problems | -0.13 | -0.18 , -0.09 | -0.09 | -0.13 , -0.05 |
| Feeling tired, exhausted, without energy | -0.51 | -0.57 , -0.45 | -0.40 | -0.44 , -0.35 |
| Problems with sleeping | -0.27 | -0.33 , -0.22 | -0.22 | -0.26 , -0.18 |
| **Functional health** |  |  |  |  |
| Assistance to walk | -0.71 | -0.86 , -0.55 | -0.43 | -0.55 , -0.30 |
| Assistance to read | -0.12 | -0.23 , -0.02 | -0.24 | -0.33 , -0.14 |
| Assistance to hear | -0.24 | -0.36 , -0.11 | -0.18 | -0.34 , -0.03 |
| **Health behaviours** |  |  |  |  |
| Smoking | -0.03 | -0.05 , -0.01 | -0.03 | -0.06 , -0.01 |
| Alcohol consumption frequency (ref. once a day and less) |  |  |  |  |
| Never | 0.08 | 0.05 , 0.12 | 0.12 | 0.09 , 0.15 |
| Twice a day | 0.08 | 0.02 , 0.14 | 0.11 | 0.02 , 0.21 |
| Three times a day | 0.01 | -0.11 , 0.14 | 0.19 | -0.08 , 0.47 |
| Physical activity in free time | 0.13 | 0.11 , 0.16 | 0.09 | 0.07 , 0.12 |
| Eating fruits daily | -0.02 | -0.07 , 0.03 | 0.05 | -0.02 , 0.12 |
| Eating vegetables daily | 0.10 | -0.00 , 0.21 | 0.21 | 0.05 , 0.38 |

Abbreviation: SRH, self-rated health

Source: Swiss Health Survey

*Form 3: “how is your health status in general? Would you say it is…”, response options: very good, good, relatively good, bad, very bad
